# Does Backgrounds Color Influence the Appearance of Gingiva-Colored Resin-Based Composites?

**DOI:** 10.3390/ma15103712

**Published:** 2022-05-22

**Authors:** María M. Pérez, Cristina Benavides-Reyes, Maria Tejada-Casado, Javier Ruiz-López, Cristina Lucena

**Affiliations:** 1 Department of Optics, Faculty of Science, Campus Fuentenueva, Edificio Mecenas, s/n., University of Granada, ibsGranada, 18071 Granada, Spain; mariatejadac@ugr.es (M.T.-C.); jruizlo@ugr.es (J.R.-L.); 2 Department of Stomatology, Faculty of Dentistry, Colegio Máximo, Campus de Cartuja s/n., University of Granada, 18071 Granada, Spain; crisbr@ugr.es (C.B.-R.); clucena@ugr.es (C.L.)

**Keywords:** gingiva-colored resin-based composite, background, color, translucency

## Abstract

Dental materials are mainly tested in vitro, so laboratory conditions must reproduce the oral environment to ensure the validity of their results. This study aimed to evaluate the influence of backgrounds on the color of gingiva-colored resin-based composites (GCRBC). Three discs of each of 20 shades of GCRBCs and each thickness (1 and 2 mm) were prepared. Diffuse reflectance was measured on-air and over three natural teeth (0M3/B1, 3M3/B3, and 5M3/B4 shades of Vita 3D Master/Vita Classical guides, respectively) using a calibrated spectroradiometer, CIE D65 illuminant and the CIE 45°/0° geometry. CIEDE2000 color difference formula and its 50:50% perceptibility and acceptability thresholds have been used to calculate and interpret the results. It can be stated that the background influences the color of all GCRBCs tested, although the effect is more pronounced for 1 mm thick samples. L*, a* and b* coordinates values of GCRBCs on air were significantly different from those obtained on natural teeth backgrounds, and the total color differences were greater than the acceptability thresholds. Since GCRBCs are placed on a dental substrate in clinical conditions, it is not advisable to perform color measurements of GCRBCs on-air because of the high color differences found. This recommendation is especially relevant for thin specimens.

## 1. Introduction

Color and appearance are determining factors that must be well-managed for success in esthetic dentistry. Research in this area has been traditionally focused on teeth and related restorative dental materials. However, an attractive smile is based on the perfect combination and harmony between both dental (white) and gingival (pink) esthetics [1].

Gingival recession is a highly prevalent pathology in the adult population [2] that often leads to esthetic concerns because of the disproportion between the height and width of the visible crown [3]. Gingiva-colored resin-based composites (GCRBC) have been proposed as a cost-effective and minimally-invasive alternative for masking the effects of gingival recession [3,4]. Although the therapeutic approach to gingival defects is usually surgical, GCRBC is a valuable option for patients with a questionable surgical prognosis or surgical contraindications [3]. Currently, a significant number of GCRBC have been introduced. However, scientific information on their performance is scarce and mainly refers to laboratory composites for indirect techniques [5,6,7]. In addition, the significant gingival color variations [8,9] by gender, age or ethnicity and the limited development of “pink esthetics” compared to the “white esthetics” lead to a persistent difficulty in mimicking the gingival color, especially in the case of lesions extending from the cervical region into the attached gingiva.

The final appearance of tooth-colored composites depends on their optical and colorimetric properties [10,11]. In addition, because of its inherent translucency, the final aspect of the restoration also depends on the color of the background (underlying tissue or air of the oral cavity) and the thickness of the restorative material [12,13,14].

Background is defined as the surface upon which samples are placed and the environment extending for about 10° from the edge of the stimulus in all or most directions [15]. The International Organization for Standardization, ISO/TR 28642:2016 [15] does not recommend using a specific background for visual assessment or instrumental color measurement. Thus, black (which, in general, represent the darkness of the oral cavity), white and grey backgrounds [16,17] have been used to evaluate color of natural teeth and to evaluate and interpret clinical outcomes and, mainly, as a quality control tool and guide to assess and select dental materials.

On the other hand, and by analogy with dental composites, it could be assumed that the GCRBC possesses a certain degree of translucency. Therefore, since they will be placed on a dental (-white) substrate, the final appearance of gingival composite restorations would result from the interaction between the color of the underlying tooth and the color, opacity, and thickness of the gingival composite [18].

Although the influence of background is often considered an important parameter for shade matching in restorative dentistry, up until now, no studies have been determining and quantifying their influence on the color of the GCRBC. The lack of scientific information on this interaction for commercially available gingival composites prevents estimating the thickness of material needed to avoid the underlying white tooth’s distorting effect and accurately reproduce the natural gingival color.

Therefore, the purpose of this study was to evaluate the influence of background’s color on the color of gingiva-colored resin-based composites, testing the null hypotheses that (1) the underlying background does not affect the color of GCRBCs, and (2) the thickness does not influence the color of the GCRBCs.

## 2. Materials and Methods

### 2.1. Preparation of Samples

The information of the materials used in this study is shown in Table 1. Three discs of each shade of each GCRCBC system and of each thickness (1 and 2 mm) were prepared using molds of Tygon^®^ tube (8 mm diameter × 1 mm/2 mm height). The mold was placed on a glass slide covered with a transparent polyester Mylar strip on the top. The resin-based composite was inserted into the mold, covered with another translucent Mylar strip, and pressed with a thin glass slide to prevent oxygen inhibition and produce a clinically relevant finish surface. Light-activation (Bluephase Style, Ivoclar-Vivadent, 1100 mW/cm^2^) was carried out by placing the 10 mm light-curing tip on the glass slide with different polymerization times according to the manufacturer specifications.

Amaris Gingiva system (Voco, Cuxhaven, Germany) consists of a base material (AMN) and three opaque shades (white (W), Light (L) and dark (D)). To prepare the AMN-W, AMN-L and AMN-D specimens, a thin and homogeneous layer of the opaque shade was placed on the bottom surface of a disc of base material (AMN) previously polymerized. Then, the opaque material was polymerized according to the manufacturer’s instructions. Reflectance measurements were performed on the opaque-free surface.

All specimens were examined for surface defects under magnification (10×). Disc thickness was verified using a digital caliper (Mitutoyo, Europe GmbH, Germany) measuring at three different specimen locations. Before spectral reflectance measurements, specimens were stored in 37 °C distilled water for 24 h in a dark chamber.

### 2.2. Color Measurement

**Figure 1:**
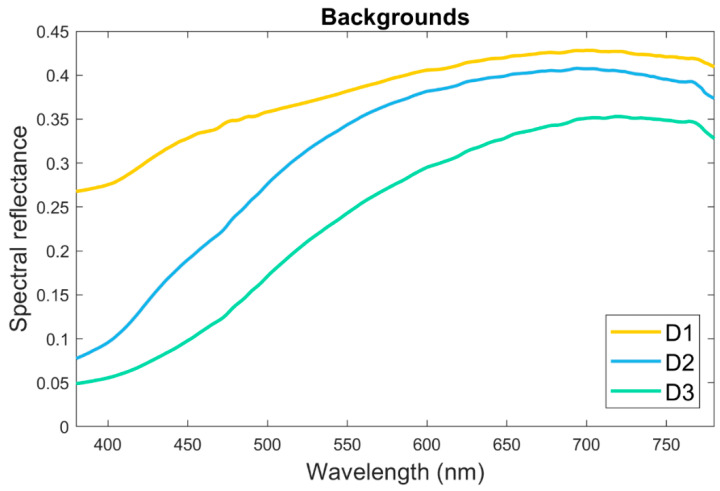
Spectral reflectance of the three natural teeth used as background.

A spectroradiometer (PR 670- Photo Research, Chatsworth, CA, USA), a fiber-coupled Xe-Arc light source (70050-300, Newport Corporation, Irvine, CA, USA) and a Spectrally Calibrated Reflectance Standard (SRS-3, Photo Research, Syracuse, NY, USA) were used to measure the spectral reflectance spectrum of the samples in the 380 nm–780 nm range, with a focus measuring aperture of 1°, at the center of each disc. The spectroradiometer was placed 40 cm away from the samples with the illuminating/measuring geometry corresponding to CIE 45°/0°. The spectral reflectance of all GCRBC specimens was measured against (i) air (i.e., without background), and (ii) three natural teeth (D1-D3) of CIELAB coordinates: Tooth 1 (D1): L* = 68.2, a* = 0.8, b* = 7.2; Tooth 2 (D2): L* = 65.0, a* = −1.0, b* = 23.2 and Tooth 3 (D3): L* = 56.6, a* = 2.3, b* = 30.1, respectively. The spectral reflectance of the three natural teeth used are represented in Figure 1.

The color of natural teeth used as backgrounds was also determined with a calibrated digital spectrophotometer (EspectroShade™ Micro, MHT, Arbizzano di Negrar, RV, Italy) as 0M3/B1, 3M3/B3, and 5M3/B4 for D1, D2, and D3, according to the VITA 3DMaster and VITA Classical shade guides, respectively.

Saturated sucrose solution having an index of refraction of approximately 1.5 was placed at the optical contact between specimen and background [19]. Three short-term repeated reflectance measurements without replacement were performed, averaged the results.

Spectral reflectance values were converted into CIE L*a*b* color coordinates using the CIE 2° Standard Observer and the CIE D65 Standard Illuminant.

The CIEDE2000 color difference Δ*E*_00_ was calculated as follows [20,21]:

(1) $$\Delta E_{00}={\left[{\left(\frac{\Delta L\prime }{k_{L}S_{L}}\right)}^{2}+{\left(\frac{\Delta C\prime }{k_{C}S_{C}}\right)}^{2}+{\left(\frac{\Delta H\prime }{k_{H}S_{H}}\right)}^{2}+R_{T}\left(\frac{\Delta C\prime }{k_{C}S_{C}}\right)\left(\frac{\Delta H\prime }{k_{H}S_{H}}\right)\right]}^{\frac{1}{2}}$$

where *k_L_S_L_*, *k_C_S_C_* and *k_H_S_H_* are correction terms used for weighting the metric differences to the CIEDE2000 differences for each coordinate. Parametric factors (*k_L_, k_C_* and *k_H_*) were set to 1 for CIEDE2000 (1:1:1).

The 50:50% perceptibility (PT) and acceptability (AT) color thresholds for human gingiva described on literature [22] (*PT*_00_ = 1.1 [95%CI 0.4–1.7] and *AT*_00_ = 2.8 [95%CI 1.8–4.0]) were used to interpret the results.

### 2.3. Statistical Analysis

After exploring the data distribution (Shapiro-Wilk and Levene’s homogeneity of varianza tests), non-parametric tests were applied. Kruskal–Wallis’s one-way analysis of variance by ranks was used to evaluate changes in thickness and between backgrounds for the color coordinates. Mann-Whitney U test with a Bonferroni correction (level of significance *p* < 0.001) was performed for pair-wise comparisons. Statistical analysis was performed using a standard statistical software package (SPSS Statistics 20.0.0, IBM Armonk, New York, NY, USA).

## 3. Results

**Figure 2:**
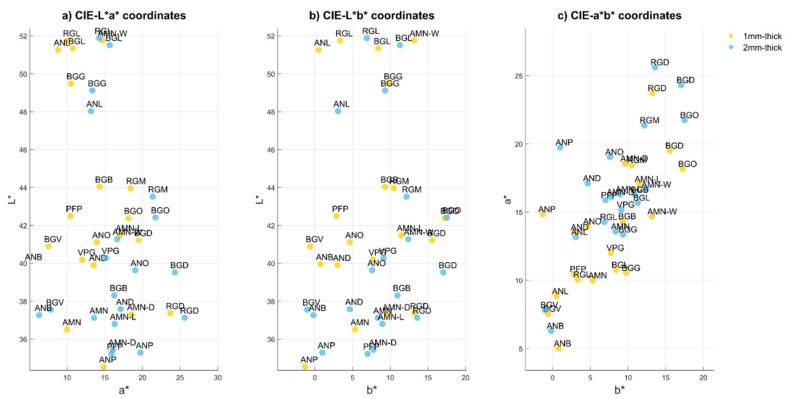
CIE L*a*b* color coordinates 2D representation of the samples measured against air: (**a**) CIE L*a* coordinates; (**b**) CIE L*b* coordinates; (**c**) CIE a*b* coordinates.

The range of color coordinates of 1 mm thickness specimens over air and on D1, D2 and D3 backgrounds were: L* = 36.5–51.8, a* = 5.0–23.7, b* = −0.6–17.3, L* = 42.4–60.0, a* = 8.9–25.5, b* = 4.6–26.7; L* = 42.0–60.8; a* = 8.7–31.2; b* = 5.3–26.9; and L* = 41.2–60.8, a* = 8.6–29.3, b* = 8.1–24.8, respectively. In addition, for 2 mm thickness specimens were: L* = 35.2–51.9, a* = 6.3–25.6, b* = −1.0–17.5; L* = 36.6–52.0, a* = 8.6–28.0, b* = 0.8–20.0; L* = 36.0–52.0, a* = 8.6–28.2, b* = 0.8–19.7; and L* = 34.4–50.2, a* = 8.4.–26.8, b* = 0.8–20.4. As an example, CIE L*a*b* color coordinates of 1 mm and 2 mm thickness specimens over air background were presented in Figure 2.

In an overall analysis, the comparison of the lightness of 1 mm thickness specimens over the different backgrounds showed a statistically significant (*p* < 0.001) between the air and the teeth backgrounds (D1–D3). However, no significant differences in lightness were found for 2 mm thickness specimens between the four backgrounds used in the study. On the other hand, the comparison of the a* and b* coordinates values between the different backgrounds showed only statistically significant (*p* < 0.001) between the air background and the teeth backgrounds (D1–D3 for both thicknesses and both coordinates).

Additionally, the comparison of the color coordinates between the two thicknesses showed statically significant differences in L* and b* for all teeth backgrounds (D1–D3) (*p* < 0.001). However, in contrast to teeth backgrounds, only the a* coordinate showed statistically significant difference between thicknesses for air background.

The mean Δ*E*_00_ values for each GCRBC and each thickness over the different backgrounds are presented in Table 2. Color differences were higher for the lower thickness (1 mm). Color differences between air background and D1/D2/D3 were above the perceptibility thresholds (*PT*_00_ > 1.1 CIEDE2000 units) for all specimens of both thicknesses. In addition, for all 1 mm specimens those Δ*E*_00_ are above acceptability thresholds (*PT*_00_ > 2.8 CIEDE2000 units). 

Δ*E*_00_ values for specimens over D1 and D2 backgrounds are not perceptible [22], except for all AnaxGUM composites and PFP, AMN, VPG, and BGB at 1 mm, which are acceptable (>*PT*_00_, ≤*AT*_00_) [22]. In general, Δ*E*_00_ values for specimens over D1 and D3 backgrounds and for both thicknesses are acceptable, and only ANP, ANP, ANM, and VPG for 1 mm thickness showed unacceptable color differences [22]. Finally, ΔE_00_ values for specimens over D2 and D3 backgrounds and for both thicknesses are acceptable (>*PT*_00_, ≤*AT*_00_), except for RGL, ANO, ANP, AMN, VPG and BGG for 1 mm thickness which are unacceptable color differences.

## 4. Discussion

The research on tooth-colored composites has shown the effect of environmental conditions, such as illumination source [5] and background color [10], on color perception. Therefore, these variables should be controlled in the experimental protocols.

The present study used the CIE 1931 2° Standard Colorimetric Observer, a light source simulating the spectral relative irradiance of CIE D65 standard, and illuminant/measuring geometry corresponding to CIE 45°/0°, to test the influence of various backgrounds (air and three natural teeth of OM3-B1, 3M-B3 and 5M3-B4 shades) on the color of different gingiva-colored resin-based composites at two clinically relevant thicknesses (1 and 2 mm).

In dentistry, most studies [23,24,25] evaluate the effect of different backgrounds by calculating the total color difference of the tested specimen through either the CIELAB or CIEDE2000 color difference formulas. However, successful esthetic restoration depends on the final appearance of the restoration and acceptable color matching with the adjacent structures rather than numerical data alone. Therefore, the interpretation of color difference values should be associated with the perceptibility (PT) and acceptability thresholds (AT) to correlate the numerical data with what is perceived and visualized clinically and assess and report the clinical significance of the results [15,26,27]. The CIEDE2000 color difference formula demonstrated a consistently better fit than CIELAB formula in evaluating color thresholds [28]. In our study, Δ*E*_00_ formula and their 50:50% perceptibility (*PT*_00_) and acceptability thresholds (*AT*_00_) [22] were used to interpret the influence of color background on the color of the GCRBC.

Based on the results of this in vitro study, it can be stated that the background influences the color of all GCRBCs tested, although the effect is more pronounced for 1 mm thick samples. Therefore, the first null hypothesis is rejected. Thus, L*a*b* coordinates values measured air background were significantly different from those obtained with the three natural teeth as backgrounds (*p* < 0.001), and the total color differences were greater than *AT*_00_ [22] (Table 2). For 2 mm thick specimens, all total color differences between air and D1–D3 backgrounds were visually perceptible (>1.1), but almost half were below *AT*_00_. However, no significant differences in lightness were found.

Regarding the influence of specimen thickness on GCBRCs color, there were statically significant differences in a* coordinate values between 1 mm and 2 mm thick specimens measured over air background. While for measurements made on teeth backgrounds (D1–D3), there were statistically significant differences for the L* and b* coordinate values. Consequently, the second null hypothesis is also rejected. These results are consistent with previous studies on tooth [29] and tooth-colored composites [24,25,29] indicating a significant background influence on color perception.

Although scientific information about the gingival composites’ translucency is not currently available, our results highlight the translucent nature of GCRBCs allows the background to show through, contributing to their final optical properties [30]. In addition, as the thickness of the samples decreases, the translucency increases [29], so the effect of background reflectance on the final specimen reflectance is more evident

The teeth used as backgrounds in our study have different reflectance curves (Figure 1) corresponding to B1, B2 and B4 Vita Classical shades. Still, they lead to low color differences between GCRBCs, mostly (94%) clinically acceptable. These results can´t be extrapolated to very dark or bright teeth as backgrounds or even to other restorative materials: a higher number of underlying tooth shades, different thicknesses of specimens should be analyzed, and further in vivo studies are necessary to confirm or reject the here obtained results

The lack of information on gingival composites’ physical, mechanical, and aesthetic properties needs a growing interest from researchers. Since most tests on dental materials are performed in the laboratory, it is essential to use the background that corresponds best to the intraoral situation and mimic the laboratory’s oral environment. According to our results, it is not advisable to perform color measurements of GCRBCs on-air (similar to a black background), because the higher color differences found whit this dark (low reflectance background). This recommendation is especially relevant for thin specimens. Composites become almost opaque as the specimen thickness approaches its infinite optical thickness, in which, regardless of the reflectance of the background, the material will express 99.9% of its inherent reflectance [31].

The present results are also clinically relevant since the background influence could lead to dramatic differences in the appearance of gingiva-colored composite resins when a thin layer of material (in the range used clinically) is superimposed on significantly darkened or brightened teeth.

## 5. Conclusions

Within the limitations of the present study, it can be stated that the background‘s color significantly influences the color of gingiva-colored resin-based composites. Since GCRBCs are placed on a dental substrate in clinical conditions, it is not advisable to perform color measurements of GCRBCs with no background (on air) in laboratory tests.

## Figures and Tables

**Table 1:** Information on the gingiva-colored resin-based composites evaluated in the study. All resin-based composites data were provided by manufacturers.

Material	Manufacturer	Shade (Code)	Batch nº	Composition	Type	Filler content wt%/vol%
**Renamel Gingafill**	Cosmedent (Chicago, IL, USA)	Light Pink **(RGL)**	1646208	Monomers: UDMA, BBDMA. Fillers: silicon dioxide and prepolymerized composite (70%), initiators, stabilizers and pigments (<1%). Particle size: 0.04–0.2 µm.	Sculptable Microfilled	70%/60%
		Medium Pink **(RGM)**	1646218			
		Dark Pink **(RGD)**	161908A			
**PermFlo^®^ Pink**	Ultradent (South Jordan, UT, USA)	Pink **(PFP)**	BH2V6	Monomers: TEGDMA, BisGMA, UDMA. Fillers: Sodium Monofluorophosphate. Particle size: 1 µm.	Flowable	68%/NC
**AnaxGUM**	Anaxdent GmbH (Stuttgard, Germany)	Light Pink **(ANL)**	2019006786	Monomers: UDMA, BDDMA, BisGMA. Fillers: anorganic fillers, pyrogenic silica, initiators, stabilizers, pigments. Particle size: 0.04–0.7 µm.	Sculptable Microfilled	74%/NC
		Dark Pink **(AND)**	2020001998			
		Orange Pink **(ANO)**	2020001526			
		Purple Pink **(ANP)**	2019006922			
		Brown Pink **(ANB)**	2011008860			
**Amaris^®^ Gingiva**	VOCO GmbH (Cuxhaven, Germany)	Natural Pink **(AMN)**	1932473	Monomers: BisGMA, UDMA, TEGDMA. Fillers: silane coated glass ceramic, pre-polymerized filler, silica nano particles.	Sculptable Nanohybrid	80%/NC
		White **(AMN-W)**	1806161	Monomers: BisGMA, UDMA, TEGDMA, HEDMA. Fillers: Catalyst	Flowable opaque Nanohybrid	
		Light Pink **(AMN-L)**	1742187			
		Dark Pink **(AMN-D)**	1745374			
**Venus Pearl Gum**	Kulzer GmbH (Hanau, Germany)	Gum **(VPG)**	K010030	Monomers: UDMA, EGDMA, TCD-DI-HEA Fillers: Barium Aluminium-boro-fluor Silicate Glass, Silica, Polymer, Titanium dioxide, fluorescent pigments, metallic oxide pigments, organic pigments, aminobenzoicacidester, BHT, Camphorquinone.	Flowable Nanohybrid	NC/59%
**Beautifil II Gingiva**	Shofu Dental (Kyoto, Japan)	Light **(BGL)**	032013	Monomers: BisGMA, TEGDMA Fillers: S-PRG Aluminium-fluor-borosilicate glass. Pigments, others	Sculptable Nanohybrid	60–70%
		Dark **(BGD)**	032012			
		Orange **(BGO)**	121904			
		Violet **(BGV)**	121904			
		Brown **(BGB)**	121905			
		Gum **(BGG)**	091916		Flowable Nanohybrid	

Abbreviations: NC: Information not collected. UDMA: urethane dimethacrylate; BBDMA: 1,4-Butanediol dimethacrylate; TEGDMA: Triethylene glycol dimethacrylate, BisGMA: bisphenol-A-glycidyldimethacrylate; HEDMA: hexanediol dimethacrylate; EGDMA: Ethylene glycol dimethacrylate, TCD-DI-HEA: 2-propenoic acid, (octahydro-4,7 methano-1H-indene-5-diyl) bis(methyleneiminocarbonyloxy-2,1-ethanediyl) ester; BHT: butylated hydroxytoluene; S-PRG: surface pre-reacted glass ionomer.

**Table 2:** Mean CIEDE2000 color difference values for each GCRBC and each thickness of all background combinations.

		A—D1	A—D2	A—D3	D1—D2	D1—D3	D2—D3
**RGL**	**1 mm**	10.7	10.4	7.3	0.8 *	3.9	3.3
	**2 mm**	2.8	2.9	2.6 **	0.1 *	2.1 **	2.1 **
**RGM**	**1 mm**	8.2	8.5	5.8	0.3 *	2.4 **	2.7 **
	**2 mm**	1.7 **	1.8 **	2.1 **	0.2 *	1.9 **	2.0 **
**RGD**	**1 mm**	7.5	7.7	5.6	0.4 *	1.9 **	2.1 **
	**2 mm**	1.4 **	1.5 **	2.0 **	0.2 *	1.6 **	1.5 **
**PFP**	**1 mm**	9.7	10.9	9.0	2.1 **	2.6 **	2.8 **
	**2 mm**	3.6	3.3	3.0	0.4 *	1.9 **	1.5 **
**ANL**	**1 mm**	9.8	10.5	8.1	1.6 **	2.6 **	2.7 **
	**2 mm**	3.1	3.3	3.3	0.3 *	2.4 **	2.3 **
**AND**	**1 mm**	11.6	12.1	9.7	2.0 **	2.7 **	2.6 **
	**2 mm**	4.4	4.4	3.7	0.2 *	2.0 **	1.9 **
**ANO**	**1 mm**	12.9	13.3	10.2	2.0 **	3.9	3.3
	**2 mm**	4.5	4.5	3.3	0.4 *	1.9 **	1.7 **
**ANP**	**1 mm**	13.9	14.7	11.8	2.8 **	4.3	3.2
	**2 mm**	3.6	3.6	3.1	0.6 *	1.9 **	1.4 **
**ANB**	**1 mm**	11.7	11.8	9.6	1.5 **	2.4 **	2.4 **
	**2 mm**	3.4	3.8	3.2	0.5 *	1.4 **	1.4 **
**AMN**	**1 mm**	16.4	17.1	13.2	2.5 **	4.5	4.3
	**2 mm**	5.3	5.3	4.0	0.3 *	2.1 **	1.9 **
**AMN-W**	**1 mm**	3.9	4.2	2.6	0.4 *	1.3 **	1.6 **
	**2 mm**	2.4 **	2.5 **	1.9 **	0.1 *	1.9 **	1.9 **
**AMN-L**	**1 mm**	6.1	6.4	4.5	0.8 *	1.6 **	2.1 **
	**2 mm**	3.6	3.8	3.0	0.3 *	1.6 **	1.6 **
**AMN-D**	**1 mm**	6.5	6.3	5.2	0.4 *	1.3 **	1.2 **
	**2 mm**	2.3 **	2.7 **	2.3 **	0.6 *	1.9 **	1.7 **
**VPG**	**1 mm**	12.7	13.3	9.8	2.0 **	3.9	3.7
	**2 mm**	3.8	3.7	2.5 **	0.3 *	2.1 **	1.9 **
**BGL**	**1 mm**	9.0	9.1	7.1	0.6 *	2.0 **	2.1 **
	**2 mm**	2.4 **	2.2 **	2.4 **	0.7 *	2.0 **	1.8 **
**BGD**	**1 mm**	9.1	9.3	7.2	1.0 *	2.2 **	2.2 **
	**2 mm**	2.1 **	2.0 **	1.5 **	0.4 *	1.7 **	1.5 **
**BGO**	**1 mm**	8.6	8.7	6.5	0.2 *	2.2 **	2.3 **
	**2 mm**	1.6 **	1.7 **	2.4 **	0.7 *	2.4 **	1.8 **
**BGV**	**1 mm**	5.9	6.3	5.0	0.7 *	1.2 **	1.5 **
	**2 mm**	2.0 **	2.2 **	2.3 **	0.2 *	1.2 **	1.2 **
**BGB**	**1 mm**	6.5	6.3	5.7	2.8 **	1.5 **	4.5
	**2 mm**	2.6 **	1.6 **	3.1	1.3 **	1.4 **	2.4 **
**BGG**	**1 mm**	11.1	11.3	9.2	0.5 *	2.0 **	2.3 **
	**2 mm**	4.1	4.2	3.8	0.1 *	2.3 **	2.3 **

Abbreviations: A: Air background; D1, D2, D3: natural teeth backgrounds; *: $\Delta E_{00}\leq PT_{00}$; **: $PT_{00}<\Delta E_{00}\leq AT_{00}$.

## Data Availability

Not applicable.
